# The Impact of the Foveal Bulge on Visual Acuity in Resolved Diabetic Macular Edema and Retinal Vein Occlusions

**DOI:** 10.7759/cureus.75543

**Published:** 2024-12-11

**Authors:** Seyi A Olaniyi, Muhammad Ali, Abhimanyu Sharma, Syeda Areeba Hussain Kazmi, Rohan Raj, Parvinder Kaur, Hamza Islam, Selim Alameddine, Mansi Singh

**Affiliations:** 1 Medicine and Surgery, Obafemi Awolowo University, Ile Ife, NGA; 2 Internal Medicine, Riphah University, Rawalpindi, PAK; 3 Medicine, Sri Guru Ram Das University of Health Sciences, Amritsar, IND; 4 Research, Johns Hopkins University School of Medicine, Baltimore, USA; 5 Medicine, Karachi Medical and Dental College, Karachi, PAK; 6 Internal Medicine, Nalanda Medical College and Hospital, Patna, IND; 7 Internal Medicine, Crimean State Medical University, Simferopol, UKR; 8 Internal Medicine, Punjab Medical College, Faisalabad, PAK; 9 Surgery, Beirut Arab University, Beirut, LBN; 10 Medicine, O. O. Bogomolets National Medical University, Kyiv, UKR

**Keywords:** diabetic eye disease, diabetic macular edema, foveal bulge, optical coherence tomography, retinal vein occlusion

## Abstract

Purpose: The purpose of this study is to evaluate the impact of foveal bulge presence on visual acuity (VA) in patients with diabetic macular edema (DME) and retinal vein occlusion (RVO).

Methods: Spectral-domain optical coherence tomography (SD-OCT) scans were conducted on 22 DME patients and 20 RVO patients. Ordinary least squares (OLS) regression was employed to analyze the association between VA and the presence of the foveal bulge, as well as factors such as sex, age, central foveal thickness, various line scans of the fovea, and the external limiting membrane (ELM).

Results: In DME patients, the β value associated with foveal bulge presence was 10.2, while the β value for ELM presence was 36.19. For RVO patients, the β value for foveal bulge presence was 18.71. In the combined analysis of DME and RVO patients, VA increased by 14.24 letters with the presence of a foveal bulge.

Conclusion: The presence of a foveal bulge significantly enhances VA in patients with resolved DME and RVO. Our findings indicate that the increase in VA is more pronounced in RVO patients compared to those with DME. Additionally, the presence of the foveal bulge, ELM, and sex can serve as predictors for VA outcomes in these patient populations.

## Introduction

Retinal vascular diseases are the leading causes of visual impairment and blindness in the Western world. Diabetic macular edema (DME) is the leading cause of vision loss among the working-age population, while retinal vein occlusion (RVO) ranks as the second most common type of retinal vascular disease [1-5]. DME is particularly detrimental for individuals with diabetes mellitus, primarily due to the disruption of the blood-retinal barrier. This disruption arises from changes in tight junctions, loss of pericytes and endothelial cells, retinal vessel leukostasis, increased vesicular transport, heightened permeability of the retinal vessels, and retinal pigment epithelium. These vascular changes contribute to the accumulation of edema within the intraretinal layers of the macula leading to impaired vision. Although the precise etiology of DME remains unclear, it has been linked to several factors, including hyperglycemia, advanced glycation end products, free radicals, protein kinase C formation, vascular endothelial growth factor (VEGF) secretion, and increased vascular permeability [6].

The foveal bulge is characterized as a protrusion at the inner segment-outer segment (IS-OS) line or ellipsoid zone at the center of the fovea, where cone density is at its maximum [7,8]. Prior studies have indicated that the presence of a foveal bulge correlates with better visual acuity (VA) in both inherited and acquired retinal disorders [8]. For instance, Hasegawa et al. found that an intact foveal bulge in patients with macular edema from branch retinal vein occlusion (BRVO) was associated with improved visual outcomes compared to those without a foveal bulge [8]. Additionally, another study by Hasegawa et al. established that the presence of a foveal bulge serves as a reliable marker for VA in patients after the repair of rhegmatogenous retinal detachment [9].

RVO involves the obstruction and dilation of the retinal venous system, leading to macular edema and potential vision loss [3,5]. This obstruction increases intraluminal pressure, resulting in transudation according to Starling’s law, which triggers a cascade of interstitial fluid accumulation and increased interstitial oncotic pressure, ultimately resulting in edema. Such edema can inhibit tissue perfusion and lead to ischemia. Ischemic events may stimulate VEGF production, exacerbating vascular permeability and worsening edema [10,11]. This cycle can significantly impair vision. Similar to DME, the exact etiology of RVO is not fully understood, though it is believed to be associated with thrombotic events. Patients with RVO often present with risk factors such as hypertension, dyslipidemia, and other vascular diseases [5].

Despite the high prevalence of DME and RVO, the precise pathogenesis of these conditions remains elusive. Furthermore, treatment options, including anti-VEGF therapy and focal/grid laser treatments, are based on mechanisms that are not fully understood [12-14]. Although these treatments have shown some effectiveness, their success largely depends on early diagnosis, timely intervention, and management of risk factors [13,15]. Consequently, regular screening for VA and related factors can serve as preventive measures. Previous research on the foveal bulge and VA suggests that the foveal bulge may function as a valuable diagnostic tool for acquired visual impairment [16]. While earlier studies have explored the relationship between the foveal bulge and VA in specific disease states, there is a notable gap in research addressing the two most prevalent retinal vascular diseases affecting a significant portion of the global population. This study aims to investigate the relationship between the foveal bulge and VA in patients with DME and RVO.

## Materials and methods

Patient eligibility and exclusion criteria

This study utilized data from the Ranibizumab for Edema of the Macula in Diabetes (READ-2) and Role of Laser in the Management of Retinal Vein Occlusion (RELATE) clinical trials [17,18]. Additionally, information from the RELATE trial with ClinicalTrials.gov identifier NCT01003106 and the READ-3 trial registered under NCT01077401 on www.clinicaltrials.gov were included in the analysis. The methodology involved a comprehensive review and analysis of the data obtained from these trials to assess the effectiveness and outcomes of the treatments in the management of macular edema and retinal vein occlusion.

Approval for this research study, which analyzed the data from the READ-2 and RELATE clinical trials, was obtained from the Johns Hopkins University School of Medicine IRB and deemed exempt. The study was conducted in compliance with the ethical standards and guidelines established by the IRB to ensure patient confidentiality, privacy, and informed consent. Data handling and analysis procedures were conducted according to the approved study protocol to uphold the ethical integrity of the research and protect the rights of the participants.

Patients with confounding ophthalmological conditions (e.g., retinal macular degeneration, severe cataracts, or prior vitrectomy), active ocular infections, media opacities affecting SD-OCT imaging, or systemic conditions (e.g., uncontrolled hypertension) were excluded to ensure a homogeneous cohort and minimize potential confounding factors.

We evaluated all the spectral domain optical coherence tomography (SD-OCT) scans of all patients who had the presence of the foveal bulge, external limiting membrane (ELM), and resolution of edema. Assessment of the resolution of DME was made by the absence of any optically clear spaces large enough for the graders to mark them as intra-retinal fluid. After evaluation of all SD-OCTs from both clinical trials, a total of 42 patients were included in our analysis for this study. All SD-OCT scans were reviewed by two independent trained graders. In case of any discrepancy in assessment between the two graders, a senior grader (SMS) reviewed and graded the SD-OCT scan.

Of the 42 patients in our analysis, 22 had resolved DME and 20 with resolved RVO. Of the 20, 10 had BRVO and 10 had CRVO. Patients with any other confounding ophthalmological factors were excluded on the basis of clinical history and physical examination findings. For each DME and RVO patient volume scan (20° x 20°; roughly 6 x 6 mm) with 25 B-scans in horizontal orientation spaced 240µm apart, minimum automatic real-time mean of 9, and high speed (512 A-scans/B-scan) was performed using Heidelberg Spectralis HRA-OCT machine (Heidelberg Engineering, Inc., Heidelberg, Germany).

**Figure 1:**
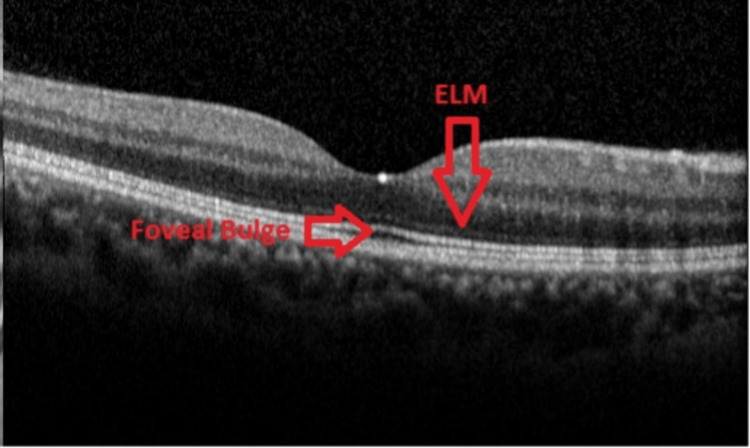
Optical coherence tomography image of a normal eye showing the location of the foveal bulge and external limiting membrane (ELM)

We evaluated the thicknesses of the central fovea, outer nuclear layer, inner segment, and outer segments of the retina as shown in Figure 1. The presence of a bulge was qualitatively determined using the SD-OCT. We defined the bulge as the central fovea up to the IS-OS line. To minimize bias, SD-OCT grading was performed in a blinded manner. In addition, we also assessed for sub-retinal fluid and external retinal membrane on horizontal cross-sectional images. Quantitatively, the foveal bulge thickness was measured as the distance (microns) between the inner border of the retina pigment epithelium and the outer border of the IS-OS line at the fovea [16].

Statistical analysis

All statistical analyses were done using StataMP 13 (StataCorp. 2013. Stata Statistical Software: Release 13, StataCorp LLC, College Station, Texas, USA). Ordinary least squares (OLS) were used to test for associations between best-corrected visual acuity (BCVA) and foveal bulge in DME, in RVO, and BCVA and foveal bulge in combined DME and RVO patients. We controlled for sex, central foveal thickness, left eye, right eye, scan cross-sections, subretinal fluids, ELM, outer nuclear layer (ONL) thickness, IS thickness, and OS thickness.

The combined analysis of DME and RVO patients assessed the relationship between foveal bulge thickness and BCVA by pooling data from both groups to form a larger dataset. This analysis utilized OLS regression, applying the same model used for individual groups while adjusting for covariates such as age, sex, and central foveal thickness to ensure consistency. The statistical analyses conducted in this study utilized OLS regression to assess the associations between BCVA and foveal bulge in patients with DME and RVO as shown in Figure 1. The p-values were calculated based on the coefficients derived from these OLS models.

The equation used in the OLS regression: $Visual Acuity = 30 + B1 &bull; FovealBulge + B2 &bull; Sex + 33 &bull; Age + B4. CentralFovealThickness + 35 &bull; StudyEye + 36 &bull; Cross - Section + B7 &bull; ELM$

Where β0 is the intercept term and β1 to β7​ are the coefficients corresponding to each independent variable.

The p-values associated with each coefficient were determined to evaluate the significance of their contributions to predicting VA. A p-value of less than 0.05 was considered statistically significant.

## Results

Patient characteristics

The average age of all patients was 63.98 years (SD = 11.42). Specifically, the average age of patients with RVO was 60.75 years (SD = 11.56), while the average age of patients with DME was 67 years (SD = 10.6). Among DME patients, the mean VA was 42.20 letters (SD = 13.64), whereas RVO patients had a mean VA of 54.50 letters (SD = 20.46). The combined mean VA for all patients (DME and RVO) was 47.73 letters (SD = 18.02) as shown in Table 1.

**Table 1:** The mean VA of all the patients (DME and RVO combined) DME: Diabetic Macular Edema, RVO: Retinal Vein Occlusion, VA: Visual Acuity, SD: Standard Deviation, OD: Oculus Dexter, OS: Oculus Sinister

Characteristics	Value
Condition	Mean Age (Years) (SD)
DME (N=22)	67.00 (10.6)
RVO (N=20)	60.75 (11.56)
Combined (N=42)	63.98 (11.42)
Condition	Female, N (%)
DME	4(18.2)
RVO	12(60)
Combined	16(38.1)
Condition	Mean VA, Score (SD)
DME	42.20 (13.64)
RVO	54.50 (20.46)
Combined	47.73 (18.02)
Condition	Study Eye= OD, OS
DME	8, 14
RVO	10, 10
Combined	18, 24

Analysis of the impact of the foveal bulge on VA in DME patients

In DME patients, the β coefficient for the presence of a foveal bulge in relation to VA was 10.14 (95% CI: 7.57-24.84, p < 0.01). The β coefficient for the ELM was 36.19 (95% CI: 18.10-54.28, p < 0.001). Other factors, including sex, age, central foveal thickness, study eye, and subretinal fluid, did not show significant correlations with VA. Specifically, VA was not significantly associated with sex (β = 6.49, p = 0.136), age (β = -0.07, p = 0.699), central foveal thickness (β = -0.01, p = 0.646), study eye (β = 1.69, p = 0.585), or subretinal fluid (β = -2.57, p = 0.63) as detailed in Table 2.

**Table 2:** β values of DME, RVO, and combined (DME + RVO) in correlation to best-corrected visual acuity (BCVA) The data has been represented with p value significant at p<0.05. DME: Diabetic Macular Edema, RVO: Retinal Vein Occlusion

	DME	p-value	RVO	p-value	Combined(DME +RVO)	p-value
Foveal Bulge	10.14	<0.010	18.71	<0.050	14.24	<0.001
Sex	6.49	0.136	1.31	0.859	10.78	<0.001
Age	-0.07	0.699	-0.37	0.189	-0.18	0.213
Central Foveal Thickness	-0.01	0.646	-0.01	0.831	0.00	0.979
Study Eye	1.69	0.585	6.36	0.333	2.10	0.500
ELM	36.19	<0.001	16.98	0.548	23.21	<0.010
Constant	10.90	0.530	27.63	0.475	25.37	0.147

Analysis of the impact of the foveal bulge on VA in RVO patients

For RVO patients, the β coefficient for the foveal bulge in relation to VA was 18.71 (95% CI: 1.76-35.65, p < 0.05). No other factors demonstrated significant correlations with VA. The presence of ELM yielded a β of 16.98 (95% CI: -41.16 to 75.11, p = 0.548), which was not significant, unlike in the DME patient population. In RVO patients, VA was also not significantly associated with sex (β = 1.31, p = 0.859), age (β = -0.37, p = 0.189), central foveal thickness (β = -0.01, p = 0.831), study eye (β = 6.36, p = 0.333), subretinal fluid (β = 6.75, p = 0.792), or the presence of ELM (β = 16.98, p = 0.548) as indicated in Table 2.

Analysis of the impact of the foveal bulge on VA in combined RVO and DME patients

In the combined analysis of DME and RVO patients, the β value for the foveal bulge in relation to VA was 14.24 (95% CI: 7.95-20.53, p < 0.001). Notably, women demonstrated significantly better VA compared to males, with a β of 10.78 (95% CI: 4.62-16.94, p < 0.010). The presence of ELM was also significantly associated with VA in the combined group, with a β of 23.21 (95% CI: 8.13-38.30, p < 0.01) as shown in Table 2.

The significance of the β values obtained in the study for the presence of a foveal bulge in DME and RVO patients highlights a strong positive association with BCVA. In DME patients, the β value for the foveal bulge was 10.14 (p < 0.01), indicating that for each unit increase in foveal bulge thickness, there is a corresponding increase in BCVA. This strong correlation suggests that both the presence and thickness of the foveal bulge are linked to better VA in these patients. Similarly, in RVO patients, the β value for the foveal bulge was 18.71 (p < 0.05), demonstrating a positive relationship between foveal bulge thickness and BCVA, which indicates that a thicker foveal bulge is associated with improved visual outcomes. When analyzing both DME and RVO patients together, the combined β value for the foveal bulge was 14.24 (p < 0.001), further reinforcing the findings and showing that a foveal bulge contributes positively to VA across both conditions. Overall, the significant β values for the foveal bulge in both patient populations underscore that the greater foveal bulge thickness correlates with better VA.

## Discussion

Our analysis demonstrates that DME patients with a pronounced foveal bulge experience better VA with an increase of 10.2 letters compared to those who lack a foveal bulge. Previous studies have indicated that a foveal bulge correlates with better VA across various retinal conditions; however, the specific relationship between foveal bulge and VA in DME patients remains inadequately understood [7,8,19]. Consistent with existing literature, our findings suggest that increased foveal bulge correlates with enhanced VA in DME patients. Additionally, the ELM was significantly linked to a 36.19-letter increase in VA, supporting the notion that ELM integrity is crucial for optimal photoreceptor function [8,20].

Hasegawa et al. found a correlation between foveal bulge presence and increased VA in patients with resolved macular edema [8]. Our study extends these findings by analyzing data from both DME and RVO patients, allowing for a robust cross-comparison and validation of our results. We also established a statistical model that may help predict VA outcomes for patients with resolved DME and RVO.

Currently, few studies focus on the impact of foveal bulge within specific retinal disease states. Hasegawa et al. noted differences in VA between control subjects and RVO patients but did not quantify the changes in BCVA. In contrast, our analysis revealed an 18.71-letter increase in VA among RVO patients with a foveal bulge, compared to a 10.14-letter increase in DME patients. This suggests that foveal bulges may have a more substantial impact on VA in RVO patients than in DME patients.

The clinical significance of the foveal bulge as a prognostic marker is underscored by its association with improved BCVA in patients with resolved macular edema. As noted, patients with a foveal bulge tend to retain better VA despite damage from macular edema. The presence of a foveal bulge may indicate a denser and thicker photoreceptor layer, which could mitigate the detrimental effects of edema. This insight may prove useful in monitoring treatment outcomes, as the presence of a foveal bulge might predict potential improvements in VA as edema resolves.

Our analysis employed OLS regression, enabling the prediction of VA based on the factors studied. The formula $\text{VA} = 10.14 \times \text{Foveal Bulge} + 6.49 \times \text{Sex} + 36.19 \times \text{ELM}$ allows for a predictive model of VA, although it is limited by the accuracy decreasing as patient age deviates from our mean age of 60.75 years. Future studies should incorporate larger sample sizes and a wider age range for more comprehensive insights.

By combining data from DME and RVO patients, we generated a heterogeneous predictive model. Even in this combined analysis, the presence of a foveal bulge resulted in a 14.24-letter increase in VA. Moreover, our findings suggest that female patients may have better VA outcomes than males, possibly due to inherent differences in visual processing abilities [21,22].

The 36-month results from the RIDE and RISE trials highlighted that sustained VA gains and anatomical improvements were achievable with monthly ranibizumab treatment for DME. Those initially assigned to sham injections showed less improvement when crossing over to treatment, emphasizing the importance of early intervention. Although both doses of ranibizumab demonstrated similar efficacy, the 0.5 mg dose raised concerns regarding serious adverse events linked to systemic VEGF inhibition [23].

In the study, the primary factors significantly influencing VA outcomes were the foveal bulge and the external limiting membrane (ELM). The foveal bulge showed a strong positive association with VA in both DME and RVO patients, while ELM was significant for DME patients but not for RVO. In our study, females showed better VA outcomes, which may be attributed to hormonal, anatomical, or genetic factors. Hormonal differences, particularly the neuroprotective effects of estrogen, may contribute to better retinal health in females. Anatomical variations, such as differences in retinal thickness or macular structure, could also play a role. Additionally, sex-linked genetic factors may influence VA outcomes [24,25]. However, other variables such as age, central foveal thickness, study eye, and the presence of subretinal fluid did not show significant correlations with VA outcomes. Overall, the focus remained primarily on these three key variables.

Future research should delve deeper into the impact of the foveal bulge on VA. While our findings confirm the role of the foveal bulge in enhancing VA, we were unable to specify which retinal layers are most influential. Further studies should explore the range and limits of the foveal bulge's impact on VA. In summary, our analysis indicates that the presence of a foveal bulge significantly enhances VA in DME patients, while also showing an even greater effect in RVO patients, with an increase of 18.71 letters. In this study, we found that the presence of a foveal bulge is significantly associated with improved VA in both DME and RVO patients, with a more pronounced effect observed in RVO. These findings highlight the potential importance of the foveal bulge as a prognostic indicator in retinal diseases. However, further research is needed to identify the specific retinal layers contributing to this improvement and to establish standardized protocols for incorporating foveal bulge assessment into routine OCT analysis for better clinical decision-making.

Limitations

This study has several limitations that should be noted. The small sample size of 22 DME patients and 20 RVO patients may affect the robustness of the findings, although we addressed this concern by checking for multicollinearity and controlling for multiple factors. Additionally, our analysis focused solely on the impact of the foveal bulge on VA in DME and RVO patients without a control group, which limits our ability to fully account for age effects on VA; however, we did control for age effects within the study population. While the specific inclusion criteria and reliance on high-quality data from established trials (READ-2, RELATE, READ-3) lend relevance to the findings, the small sample size limits statistical power, increases the risk of Type II errors, and reduces generalizability to the broader DME or RVO populations. Furthermore, fewer patients mean that outliers can disproportionately affect results. Overall, while the findings are credible, further research with larger cohorts is essential for confirmation.

## Conclusions

In conclusion, our analysis reveals that the presence of a foveal bulge is significantly associated with improved VA in patients with resolved DME and RVO. Notably, RVO patients demonstrate a greater increase in VA compared to those with DME. Additionally, factors such as the presence of the foveal bulge, ELM integrity, and the patient's sex may serve as valuable predictors for VA outcomes in these individuals. These findings highlight the importance of considering these factors in the management and prognostication of patients with resolved DME and RVO, ultimately guiding clinical decision-making and optimizing visual outcomes.
